# A Combination of Pre- and Post-Exposure Ascorbic Acid Rescues Mice from Radiation-Induced Lethal Gastrointestinal Damage

**DOI:** 10.3390/ijms141019618

**Published:** 2013-09-27

**Authors:** Yasutoshi Ito, Manabu Kinoshita, Tetsuo Yamamoto, Tomohito Sato, Takeyuki Obara, Daizoh Saitoh, Shuhji Seki, Yukihiro Takahashi

**Affiliations:** 1 Military Medicine Research Unit, Test and Evaluation Command, Ground Self-Defense Force, 1-2-24 Ikejiri, Setagaya, Tokyo 154-8566, Japan; E-Mails: hosp1-teb@inet.gsdf.mod.go.jp (Y.I.); hosp1-teb@inet.gsdf.mod.go.jp (T.Y.); dr21043@ndmc.ac.jp (T.S.); hosp1-teb@inet.gsdf.mod.go.jp (T.O.); hosp0-teb@inet.gsdf.mod.go.jp (Y.T.); 2 Department of Immunology and Microbiology, National Defense Medical College, 3-2 Namiki, Tokorozawa, Saitama 359-8613, Japan; E-Mail: btraums@ndmc.ac.jp; 3 Division of Traumatology, Research Institute, National Defense Medical College, 3-2 Namiki, Tokorozawa, Saitama 359-8613, Japan; E-Mail: ds0711@ndmc.ac.jp

**Keywords:** ascorbic acid, abdominal radiation, citrulline, radiation-induced gastrointestinal damage, post-irradiation treatment with ascorbic acid

## Abstract

The development of an effective therapy for radiation-induced gastrointestinal damage is important, because it is currently a major complication of treatment and there are few effective therapies available. Although we have recently demonstrated that pretreatment with ascorbic acid attenuates lethal gastrointestinal damage in irradiated mice, more than half of mice eventually died, thus indicating that better approach was needed. We then investigated a more effective therapy for radiation-induced gastrointestinal damage. Mice receiving abdominal radiation at 13 Gy were orally administered ascorbic acid (250 mg/kg/day) for three days before radiation (pretreatment), one shot of engulfment (250 mg/kg) at 8 h before radiation, or were administered the agent for seven days after radiation (post-treatment). None of the control mice survived the abdominal radiation at 13 Gy due to severe gastrointestinal damage (without bone marrow damage). Neither pretreatment with ascorbic acid (20% survival), engulfment (20%), nor post-treatment (0%) was effective in irradiated mice. However, combination therapy using ascorbic acid, including pretreatment, engulfment and post-treatment, rescued all of the mice from lethal abdominal radiation, and was accompanied by remarkable improvements in the gastrointestinal damage (100% survival). Omitting post-treatment from the combination therapy with ascorbic acid markedly reduced the mouse survival (20% survival), suggesting the importance of post-treatment with ascorbic acid. Combination therapy with ascorbic acid may be a potent therapeutic tool for radiation-induced gastrointestinal damage.

## 1. Introduction

Bone marrow failure is the most common complication following acute radiation exposure, and hematopoietic stem cell transplantation has been established as an effective therapeutic approach for the condition [1,2]. In contrast, there are few effective therapies for radiation-induced gastrointestinal (GI) damage, despite the fact that the GI tract is also known to be highly vulnerable to radiation-induced damage [3]. Therefore, the development of an effective treatment for radiation-induced GI damage is crucial for the intensive care of victims with high-dose radiation exposure. We previously reported that pretreatment of mice with ascorbic acid can attenuate radiation-induced lethal GI damage [4]. In that study, we orally treated the mice with ascorbic acid for three days before lethal whole body irradiation at 14 Gy, followed by bone marrow transplantation one day after radiation. Nevertheless, we were able to rescue less than half of the mice from the radiation exposure in that model (40% survival).

Radiation-induced GI damage sometimes occurs in patients receiving abdominal irradiation to treat an abdominal malignancy [5,6]. Although these patients complain of pernicious abdominal pain and/or diarrhea, resulting in impaired absorption of nutrients and malnutrition, there have been few effective therapies developed so far. Therefore, an effective therapy for radiation-induced GI damage is highly anticipated, and could be helpful for both accidental radiation exposure and for exposure to radiation with a therapeutic intent.

Focusing on the development of a treatment for GI syndrome, we used an abdominal irradiation model in the present study that was not complicated with bone marrow damage. Therefore, this model does not require a murine sacrifice as a donor of bone marrow cells, and is able to simplify the effects of the injury and treatment. In addition, abdominal irradiation is frequently performed on patients suffering from abdominal malignancy. It is therefore rational to research and develop an effective therapy for radiation-induced GI damage using a model of abdominal radiation.

In the present study, we modified the administration of ascorbic acid to enhance its radio-protective potential. We examined therapy using ascorbic acid treatment via daily oral intake and one shot engulfment (boosting) as a pretreatment. Although post-treatment alone was ineffective in our previous study [4], we herein evaluated the effects of post-treatment with ascorbic acid following radiation exposure, together with the ascorbic acid pretreatment. As a result, we were able to elicit a remarkable radio-protective effect against radiation-induced lethal GI damage (100% survival). The present therapy using pre- and post-exposure ascorbic acid may represent a potent therapeutic tool against radiation-induced GI damage. We also evaluated the GI damage by examining the plasma citrulline levels, because the level can reportedly serve as a biomarker of intestinal failure due to the enterocyte mass reduction [7]. We confirmed that the plasma citrulline level may therefore be an effective biomarker for radiation-induced GI syndrome.

## 2. Results and Discussion

### 2.1. Results

#### 2.1.1. Mouse Survival after Abdominal Irradiation

**Figure 1:**
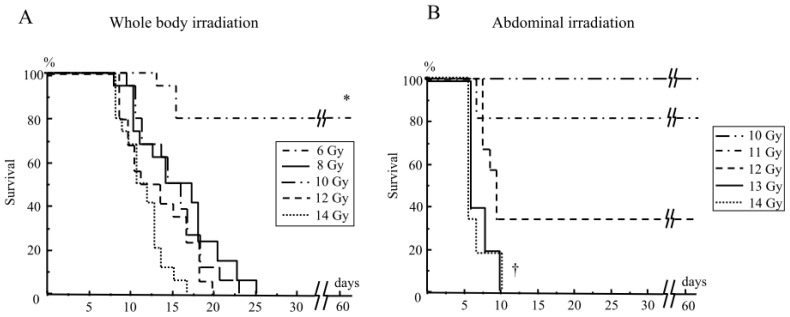
The survival of mice after whole body irradiation (**A**) and abdominal irradiation (**B**). Mice received whole body irradiation at 6–14 Gy (**A**) or abdominal irradiation at 10–14 Gy (**B**). *N* = 15 in each group, * *p* < 0.01 *vs.* others, ^†^*p* < 0.01 *vs.* 10, 11, and 12 Gy abdominal irradiation.

Mice received either whole body irradiation with 6–14 Gy or abdominal irradiation with 10–14 Gy. Although no mice survived after whole body irradiation at the doses of 8 Gy and above (Figure 1A), all mice survived after abdominal irradiation, even at 10 Gy (Figure 1B). However, no mice survived after abdominal irradiation at 13 Gy and above (Figure 1B).

#### 2.1.2. Damage to the Mouse Bone Marrow and Gastrointestinal Tract after Abdominal Irradiation

**Figure 2:**
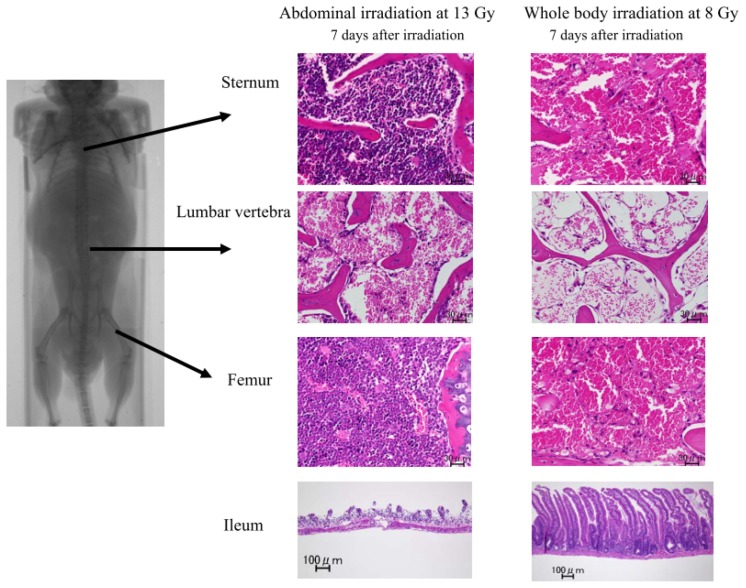
The pathological findings of the bone marrow and ileum in the mice after abdominal irradiation at 13 Gy or whole body irradiation at 8 Gy. Seven days after irradiation, the sternum, lumbar vertebra, femur and ileum were obtained from the irradiated mice, and the radiation-induced damage to these organs was examined. The images shown are representative of each group (*n* = 5), ×40 H. E.

Although the mice receiving whole body irradiation at 8 Gy showed severe bone marrow aplasia in the lumbar vertebra, the sternum and the femur seven days after irradiation (Figure 2), the mice receiving abdominal irradiation at 13 Gy still had a substantial amount of bone marrow cells in the sternum and the femur (Figure 2), suggesting that the mice had persistent bone marrow function even after lethal abdominal irradiation at 13 Gy. However, their lumbar vertebra showed severe aplasia (Figure 2), because this part was exposed to substantial doses during the abdominal irradiation.

The mice receiving abdominal irradiation at 13 Gy showed marked denudation of the gastrointestinal mucosa, especially the ileac mucosa, seven days after irradiation (Figure 2). They also showed marked denudation in the gastric, jejunal and cecal mucosae compared to those in the normal mice (Figure S1). In contrast, the mice receiving whole body irradiation at 8 Gy did not show such severe intestinal damage (Figure 2), although they all eventually died, presumably due to severe bone marrow aplasia (Figure 1A). These findings suggest that abdominal irradiation induces severe gastrointestinal damage, but does not induce extensive bone marrow damage in mice.

#### 2.1.3. Changes in the Hematological Parameters and Plasma Citrulline Levels after Abdominal Irradiation

**Figure 3:**
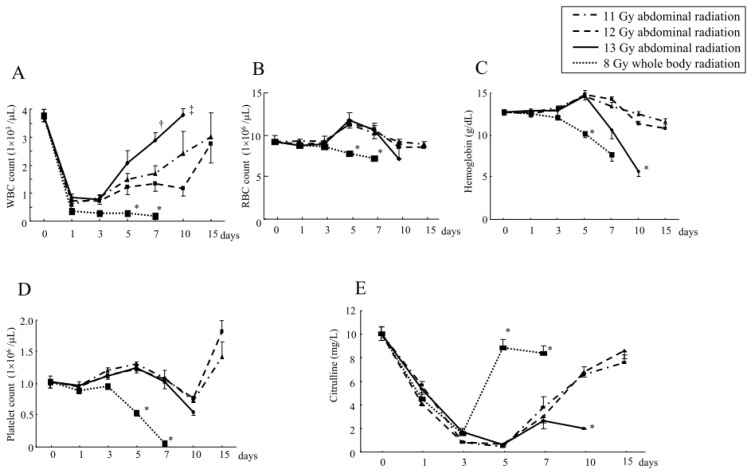
The changes in the hematological parameters and plasma citrulline levels after abdominal irradiation. Mice received abdominal irradiation at 11, 12 or 13 Gy or whole body irradiation at 8 Gy. Then, their white blood cell (WBC) count (**A**), red blood cell (RBC) count (**B**), hemoglobin level (**C**), platelet count (**D**) and plasma citrulline levels (**E**) were measured at the indicated times. The data are the means ± SE from *n* = 5 in each group, * *p* < 0.01, ^†^*p* < 0.05 *vs.* others and ^‡^*p* < 0.01 *vs.* 12 Gy.

Despite transient decreases one to three days after irradiation, the WBC counts were increased in mice beyond five days after abdominal irradiation at 11, 12 and 13 Gy (Figure 3A). In contrast, the mice receiving whole body irradiation at 8 Gy did not show such a restoration of the WBC counts and eventually died, suggesting that there had been irreversible lethal bone marrow damage (Figure 3A). The RBC counts, as well as the Hb levels, were also increased around five to seven days after abdominal irradiation at 11 and 12 Gy (Figure 3B,C), although such an increase in the RBC counts or Hb levels were not observed in the mice receiving 8 Gy whole body irradiation (Figure 3B,C). However, the mice receiving abdominal irradiation at 13 Gy showed severe anemia, as assessed by the RBC counts and Hb levels at 10 days after irradiation due to gastrointestinal bleeding (Figure 3B,C) and they subsequently died (Figure 1B). The platelet counts had not obviously decreased after abdominal irradiation at 11, 12 and 13 Gy, but markedly decreased after whole body irradiation at 8 Gy (Figure 3D). These findings suggest that abdominal irradiation even at 13 Gy (which is lethal) does not cause severe suppression of the bone marrow cells in mice.

Although the plasma citrulline levels in mice were decreased around three to seven days after abdominal irradiation at 11 and 12 Gy, they were recovered beyond 10 days after the radiation exposure (Figure 3E). However, the mice receiving abdominal irradiation at 13 Gy did not show restoration of the citrulline levels at 10 days (Figure 3E). Since their intestinal mucosae were also severely impaired at that point (Figure 2), the plasma citrulline levels after abdominal irradiation may reflect the change in the intestinal degradation. In line with this finding, the mice receiving whole body irradiation at 8 Gy showed significant restoration of the plasma citrulline levels at five to seven days (Figure 3E) without showing intestinal damage (Figure 2).

#### 2.1.4. Treatment with Ascorbic Acid before and after Abdominal Irradiation

**Figure 4:**
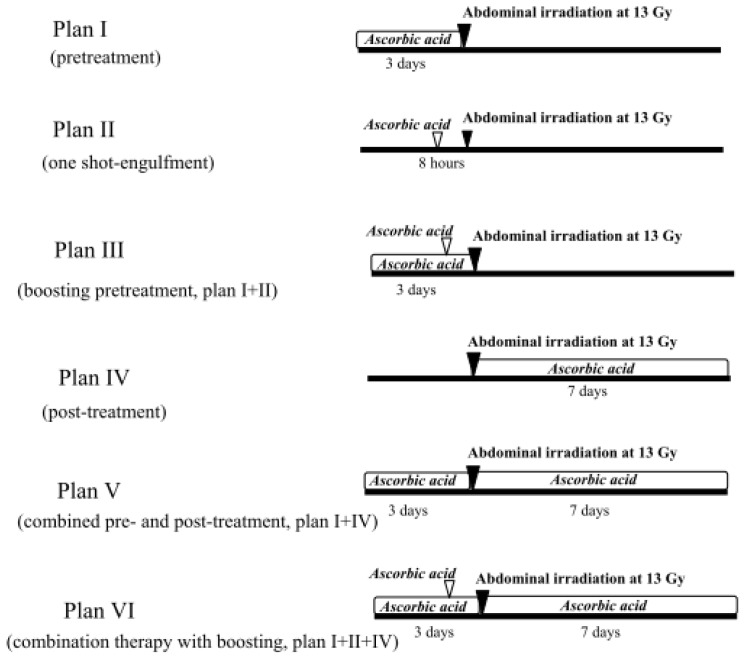
The experimental design for the abdominal irradiation and the treatments with ascorbic acid.

**Figure 5:**
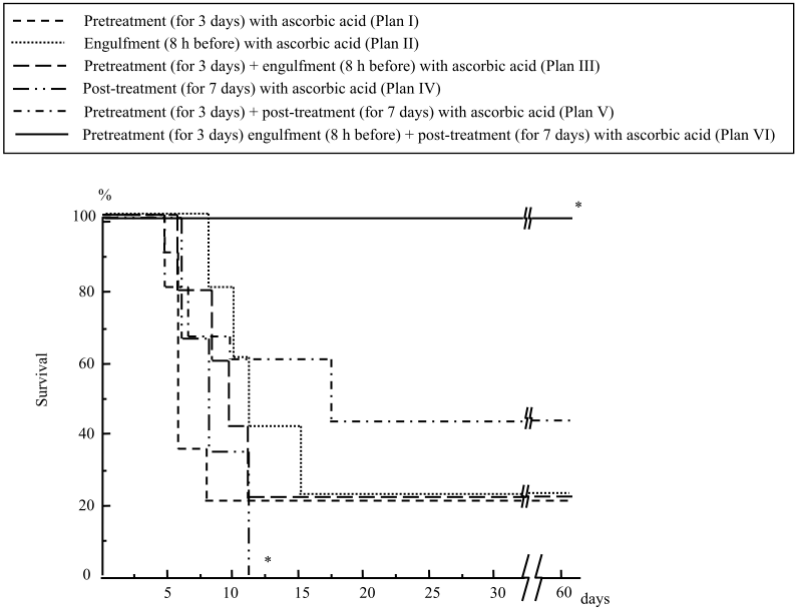
The effects of treatment with ascorbic acid on the survival of mice receiving abdominal irradiation. The mice received various treatments with ascorbic acid before/after radiation. Then they received abdominal radiation at 13 Gy. *N* = 15 in each group, * *p* < 0.01 *vs.* others.

The oral administration of ascorbic acid for three days before irradiation (Plan I, Figure 4) rescued only 20% of the mice from the lethal abdominal irradiation at 13 Gy (Figure 5). Engulfing ascorbic acid administered eight hours before radiation (Plan II, Figure 4) also rescued 20% of the mice from abdominal irradiation at 13 Gy (Figure 5). We also examined the engulfment of ascorbic acid two hours before radiation exposure in the mice; however, their survival rate was less than 10% (data not shown).

Next, we performed the boost pretreatment of mice by oral administration of ascorbic acid for three days and its engulfment eight hours before radiation (Plan III, Figure 4); however, the mouse survival rate was still 20% (Figure 5). Although post-treatment with oral administration of ascorbic acid was ineffective (0% survival, Figure 5), we tried the combination of treatment before and after radiation in mice (Plan V, Figure 4). Consequently, the survival rate increased to 40% (Figure 5). Moreover, when we added the one shot engulfment (eight hours before radiation) of ascorbic acid in addition to the oral administration for 10 days before/after radiation (Plan VI, Figure 4), the mouse survival was remarkably increased (100% survival) after abdominal radiation (Figure 5).

#### 2.1.5. Intestinal Tissue and Plasma Levels of Ascorbic Acid in Mice Treated with Ascorbic Acid

Pretreatment with ascorbic acid for three days before radiation significantly increased the ascorbic acid levels in the intestinal tissue just before radiation in comparison to that of the untreated mice (Table 1). The administration of engulfing ascorbic acid eight hours before radiation also tended to increase the tissue ascorbic acid levels, but not significantly (Table 1). The boosting pretreatment with ascorbic acid (oral intake for three days and engulfment at eight hours before radiation) further increased the tissue ascorbic acid levels in the intestine just before radiation (Table 1). However, there were no significant differences in the plasma levels among these mouse groups (Table 1).

#### 2.1.6. Intestinal Damage, Plasma Citrulline Levels, Plasma and Intestinal Tissue Levels of Free Radical Metabolites after Abdominal Radiation in Mice Treated with Ascorbic Acid

**Figure 6:**
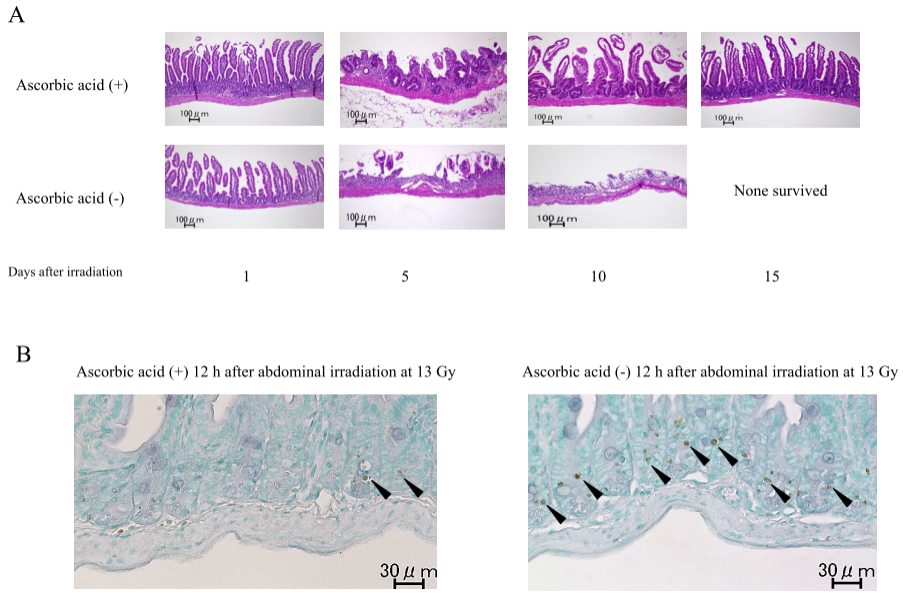
The changes in the intestinal mucosa of the irradiated mice with or without ascorbic acid treatment. Mice were treated with or without the combination therapy with ascorbic acid (Plan VI, Figure 4) and received abdominal irradiation at 13 Gy. Small intestine samples were obtained from the mice at the indicated times, and were stained with H. E. (**A**) or TUNEL (**B**) stain. The arrows indicate cells that were positive for TUNEL staining. The images shown are representative of each group (*n* = 5), ×40.

The treatment with the oral administration of ascorbic acid for 10 days before/after radiation and one shot engulfment at eight hours before radiation (Plan VI, Figure 4) significantly restored the intestinal damage in mice at 10 and 15 days after abdominal irradiation at 13 Gy (Figure 6A). This combination therapy with ascorbic acid also suppressed the positive TUNEL staining in the ileac mucosa in mice 12 h after abdominal irradiation at 13 Gy (Figure 6B), suggesting that ascorbic acid suppressed the radiation-induced apoptosis in the intestinal mucosa.

**Figure 7:**
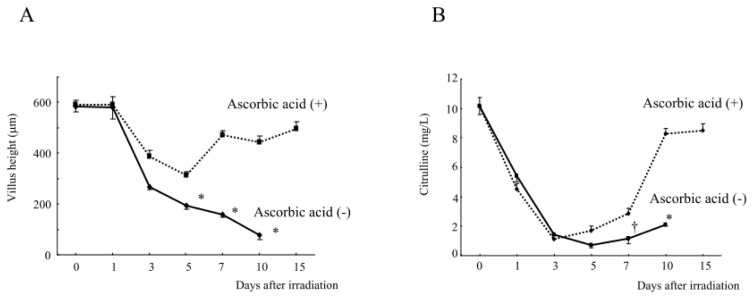
The changes in the villus height (**A**) and plasma citrulline levels (**B**) in the irradiated mice with and without ascorbic acid treatment. Mice were treated with or without the combination therapy with ascorbic acid (Plan VI, Figure 4) and received abdominal irradiation at 13 Gy. Mice were sacrificed at the indicated times to obtain the small intestine and measure its villus heights (**A**). Blood samples were also obtained from the sacrificed mice to measure the plasma citrulline levels (**B**). The data are the means ± SE from *n* = 5 in each group, * *p* < 0.01, ^†^*p* < 0.05 *vs.* other.

Consistently, the treatment with pre/post and engulfment ascorbic acid restored the villus height of ileac mucosa beyond seven days after abdominal irradiation (Figure 7A), and also restored the plasma citrulline levels (Figure 7B), suggesting that there is a relationship between the plasma citrulline levels and intestinal damage. This regiment of ascorbic acid treatment may potently improve the radiation-induced intestinal damage, thereby increasing the survival of mice after lethal abdominal irradiation.

The treatment with pre/post and engulfment ascorbic acid also significantly suppressed the free radical metabolite levels in the small intestines in mice at seven days after abdominal irradiation at 13 Gy (Table 2). However, the treatment did not affect the plasma levels of free radical metabolites in the irradiated mice (Table 2), although both mouse groups tended to have increased free radical metabolite levels in the plasma after abdominal radiation (before radiation; 167 ± 7 UCarr). Treatment with ascorbic acid also significantly suppressed the TNF levels in the small intestine and tended to suppress the plasma TNF levels in the irradiated mice at seven days (Table 2). However, there were no significant differences in the intestinal tissue/plasma IL-1β or IL-6 levels between the irradiated mice treated with and without ascorbic acid (Table 2).

### 2.2. Discussion

Pretreatment with ascorbic acid is believed to be effective for attenuating radiation-induced GI damage because of its potent antioxidative effect [8]. It is considered that the production of reactive oxygen species (ROS) is evoked immediately after radiation exposure, and that ascorbic acid may scavenge these ROS. In line with this theory, post-treatment with ascorbic acid alone (without pretreatment) was ineffective (0% survival, Figure 5), likely because it could not scavenge the ROS generated immediately after irradiation. However, even when we performed pretreatment with ascorbic acid for three days and engulfment eight hours before the irradiation, the irradiated mice showed only 20% survival (Figure 5), despite the fact that this boosting pretreatment significantly increased the tissue ascorbic acid levels just before irradiation (Table 1).

Nevertheless, the addition of post-treatment ascorbic acid to the pretreatments increased the survival of the irradiated mice to 100%. It is possible that scavenging the ROS generated immediately after radiation by the boosting pretreatment with ascorbic acid may be necessary to improve the survival of irradiated mice. However, additional post-treatment with ascorbic acid also may be indispensable for further improving the survival due to late or ongoing damage.

In our previous study [4], we examined the efficacy of ascorbic acid therapy in irradiated mice at a dose of 150 mg/kg/day. However, most clinical studies of high-dose ascorbic acid therapy, which were performed to examine the effects of ascorbic acid on the prognoses in cancer patients, were evaluated by p.o. supplementation of ascorbic acid at 10 g/day [9,10]. Therefore, we based our dosing on this level (200–250 mg/kg/day).

It is known that plants and most animals, including mice, can synthesize ascorbic acid from glucose. Primitive fish, amphibians and reptiles synthesize ascorbic acid in the kidneys, whereas most mammals, such as mice, synthesize it in the liver. In contrast, humans, other primates, guinea-pigs and a few species of fruit-eating bats cannot synthesize ascorbic acid because the gene encoding l-gulonolactone oxidase (GLO)—the enzyme required for the last step in ascorbate synthesis—is not functional [11]. The ascorbic acid concentrations in such mammals appear to be affected by three mechanisms, particularly in the human; intestinal absorption of the ingested ascorbic acid, tissue accumulation and renal reabsorption and excretion [9,12]. We think that the mice receiving high-dose ascorbic acid treatment likely have an ascorbic acid metabolism which is similar to that in humans. The gastrointestinal absorption of ascorbic acid usually occurs through an active transport process, as well as through passive diffusion. Although the active transport of ascorbic acid predominates at low intra-intestinal tract concentrations, when the hosts receives a high oral dose of ascorbic acid and develops high intra-tract concentrations (as would be expected in the present model), active transport becomes saturated, leaving only passive diffusion. In such a case, the intestinal tissue levels of ascorbic acid are important, and this is the case for the hosts receiving p.o. therapy with high-dose ascorbic acid.

The half-life of the radicals evoked by radiation is considered to be extremely short (nanoseconds), and these radicals interact with various biological molecules, with some breaking DNA chains [13]. It has been believed that these short-lived radicals play crucial roles in modulating the radiation-induced biological effects on the tissue and cells, such as apoptotic cell death [14]. When the radiation hits the water molecules in cells, which are composed of more than 80% water, it leads to the prompt generation of radicals of water origin, such as H and OH, resulting in ROS-induced tissue damage [14]. However, a cell is not a homogenous water solution of organic matter, and almost half of the water in cells exists as bound water [15]. Therefore, it is doubtful that the active radicals derived from the ionization water molecules directly participate in cell death [16].

Miyazaki *et al.* have identified long-lived radicals, which have a half-life of more than 20 h, in γ-irradiated cells, using electron spin resonance (ESR) spectroscopy and highly sensitive measurement techniques [17]. Interestingly, ESR spectroscopy showed that when ascorbic acid is added after irradiation, it removes the long-lived radicals from irradiated cells [17]. In another *in vitro* study, they demonstrated that the long-lived radicals induced by ionizing radiation are highly mutagenic and transforming in mammalian cells, although they are not involved in the lethality or in the induction of chromosome aberrations in cultured cells [16]. When ascorbic acid was added 20 h after irradiation, it reduced the frequency of radiation-induced hypoxanthine-guanine phosphoribosyltransferase (HPRT) mutation in human embryonic (HE17) cells. However, ascorbic acid did not change the survival of the cultured cells. The cell damage caused by the long-lived radicals may be limited, because suppressing the short-lived radicals by pretreatment with ascorbic acid clearly increased the cell survival [16]. In the present study, there is a possibility that the additional treatment with ascorbic acid following the pretreatment period may have effectively reduced the levels of long-lived radicals in the irradiated mice. However, we could not detect the long-lived radicals induced by irradiation in mice, and therefore, did not examine whether the long-lived radicals are scavenged by post-treatment with ascorbic acid. There is plenty of room for further research on other possible mechanisms by which the combination therapy with ascorbic acid induces a beneficial effect on the survival of irradiated mice.

Proinflammatory cytokines, as well as ROS, may play important roles in the occurrence of radiation-induced injury [18–20]. The levels of proinflammatory cytokines, e.g., TNF in the intestinal tissue (but not plasma) and IL-6 in the plasma (but not intestinal tissue), are reportedly increased in mice three to six days after abdominal radiation [18]. Mice receiving repeated abdominal radiation also reportedly exhibit a positive expression of TNF (but not IL-1β or IL-6) in the intestinal mucosa on immunohistochemistry [19]. Interestingly, in the present study, pre/post-treatment and engulfment of ascorbic acid significantly reduced the tissue TNF levels and free radical metabolite levels in the murine small intestine (but not plasma) seven days after abdominal radiation, although the treatment affected neither the IL-1β nor IL-6 levels in the intestinal tissue or plasma (Table 2). Treatment with ascorbic acid may reduce ROS and TNF-induced/involved tissue inflammatory responses, especially in the intestinal tissue, resulting in the attenuation of radiation-induced tissue injury. Further studies are required to determine how ascorbic acid therapy affects radiation-induced inflammation and cytokine responses.

The antioxidant *N*-acetyl-cysteine (NAC) reportedly protected mice from GI damage when the administration was started not only 4 h before, but also 2 h after, a lethal level of abdominal radiation [21]. Although half of the irradiated mice receiving post-treatment with NAC still died (50% survival), its pharmaceutical efficacy when administered after exposure is attractive. On the one hand, the intravenous or oral administration of ascorbic acid, including high-dose administration, is widely used in humans and is believed to be harmless because ascorbic acid is a hydrosoluble vitamin [9,10]. On the other hand, NAC has also been used intravenously and orally in humans [22–24]. However, clinicians must pay attention to the possibility of anaphylactoid reactions when intravenously infusing NAC [22]. Kerr *et al.* reported that approximately 15% of patients treated with intravenous NAC develop an anaphylactoid reaction within two hours after the initial infusion [25]. AEOL 10150, a small-molecule antioxidant analogous to the catalytic site of superoxide dismutase (SOD), reportedly protects cells against radiation-induced lung injury, even when administered after radiation exposure to the right hemithorax [26]. Although AEOL 10150 is reportedly undergoing evaluations in various clinical trials [27], it is not yet available in the clinical setting. Antioxidative agents can be potent therapeutic tools for radiation-induced tissue/organ damage.

Although abdominal irradiation is performed in patients as an effective therapy for several kinds of abdominal malignancies, radiation-induced GI damage sometimes occurs, which decreases the quality of life for the patients and treatment may need to be discontinued. The present pre/post-irradiation combination therapy with ascorbic acid may provide an effective therapeutic tool for the patients who develop radiation-induced GI damage. In addition, monitoring the plasma citrulline levels may be useful to evaluate the radiation-induced GI damage, especially after abdominal irradiation [28], because decreases in the plasma citrulline levels were associated with the intestinal mucosal damage after abdominal radiation.

Considering the potential use of ascorbic acid therapy in cancer patients, we were concerned about the possibility that the radioprotective effect induced by the ascorbic acid might reduce the radiation-induced antitumor cytotoxicity. Of note, it is known that ascorbic acid has some anticancer effects [9,12,29,30], although this was not confirmed by the clinical trial performed at the Mayo Clinic [10]. Interestingly, ascorbic acid therapy in mice reportedly exerted radioprotective effects on their skin and bone marrow cells, but did not protect the tumor cells, although the mechanism underlying this differential radiomodification of the normal tissue sensitivity and tumor tissue response is unclear [31]. A recent paper has also demonstrated that ascorbic acid enhances the radiation-induced apoptosis in tumor cell lines via the activation of caspases-3, 8 and 9 [32]. Although the precise mechanisms underlying this effect have yet to be elucidated, there is a possibility that ascorbic acid may not reduce the radiation-induced antitumor activity. However, further studies are needed to elucidate this important issue.

When a radiation accident unfortunately occurs, it is quite difficult to treat the victims with acute radiation syndrome. However, rescue team members can be deployed in radiation-contaminated areas to rescue the victims. In such situations, preventing radiation-induced damage, including GI damage, is of vital importance. As described in our previous paper [4], when rescuing victims from radiation-contaminated areas immediately after a radiation accident or terrorist attack, it is important for the rescue team members to promptly take ascorbic acid orally. The Fukushima nuclear disaster occurred in 2011, one year after the publication of our paper. This was an opportunity for our rescue team members to administer the appropriate mitigating treatment based on our scenario. Some of the volunteers in the rescue team orally took ascorbic acid as a trial, but it showed no significant side effects; however, they were fortunately considered to have received very little radiation exposure.

## 3. Experimental Section

This study was conducted according to the guidelines of the Institutional Review Board for the Care of Animal Subjects at the National Defense Medical College, Japan.

### 3.1. Mice and Reagents

Male C57BL/6 mice (20 ± 2 g body weight) were purchased from Japan SLC (Shizuoka, Japan) and used when they were eight weeks old. Ascorbic acid was purchased from Wako (Osaka, Japan).

### 3.2. Experimental Animals and Abdominal X-Ray Irradiation

**Figure 8:**
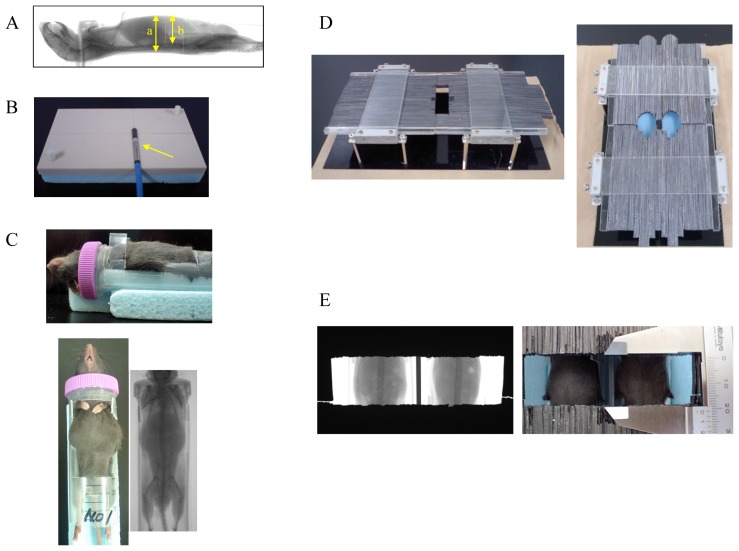
The device used for abdominal irradiation in mice. The measurement of the mouse body thickness using an X-ray image, a: the body thickness, b: the depth of the abdominal cavity (**A**); A special water equivalent phantom for mice and a radiation dosimeter, indicated by an arrow (**B**); A mouse was placed in a special container to allow for irradiation of its abdomen (**C**); A lead protector consisting of lead strips was used to administer precise irradiation (**D**, **right**); The irradiated area was adjusted by moving the lead strips (**D**, **left**) and was confirmed using X-rays (**E**).

We first measured the body thickness in mice using an X-ray image and found that it was approximately 18 ± 1 mm (Figure 8A, indicated by arrow “a”). The GI tract was also located within 13 mm from the surface of the mouse abdomen (Figure 8A, arrow “b”). To examine the precise dose of radiation exposure to the mouse abdomen, we made a special water equivalent phantom modified to match the mouse body thickness (18 mm) (Tough water WD, Kyoto Kagaku, Kyoto, Japan) (Figure 8B). We next measured the radiation dose at a point 6 mm below the surface of the simulated mouse abdomen (anterior-posterior) using a radiation dosimeter (Semiflex Ionization Chamber 31013, PTW Co., Freiburg, Germany) (Figure 8B, arrow), which was inserted in the phantom, and then adjusted the respective radiation doses (at 6–14 Gy) provided by the X-ray irradiator (MBR-1520R-3, HITACH, Tokyo, Japan).

In the animal experiments, the mice were placed in a specially designed plastic container that tightly fixed their neck and groin (Figure 8C). Subsequently, the mice were covered with a protector consisting of lead strips (a piece of strip: 1 mm in width, 5 mm in thickness) to ensure that the mouse abdomen was irradiated, while avoiding the exposure of other parts to radiation (Figure 8D, left). We confirmed that this thickness of the lead protector (5 mm) was sufficient to protect against radiation exposure by the X-ray irradiator (HITACH MBR-1520R-3). Using this protector, the irradiated area could be adjusted to various sizes and shapes by moving the lead strips (Figure 8D, right). After placing the mice in the plastic container and covering them with the lead protector, we adjusted the exposure area to the Th12–L5 levels in mice using an X-ray image (Figure 8E). Thereafter, their abdomens were exposed to radiation, given at a dose rate of 4.9 Gy/min at 150 kV and 20 mA (HITACH MBR-1520R-3). The beam was filtered through a 2 mm aluminum board.

### 3.3. Administration of Ascorbic Acid

The mice either received a *per os* (p.o.) administration of ascorbic acid that had been dissolved in distilled water at 250 mg/kg/4 mL/day for three days before abdominal irradiation (Plan I, Figure 4), or an engulfment with 250 mg/kg/0.1 mL of ascorbic acid using mouse feeding needles eight hours before irradiation (Plan II, Figure 4). Mice also received a “combination therapy” with the boosting pretreatment with ascorbic acid by oral intake for three days (250 mg/kg/day) and one shot engulfment (250 mg/kg) eight hours before irradiation (Plan III, Figure 4). Post p.o. treatment with ascorbic acid (250 mg/kg/4 mL/day for seven days after abdominal irradiation) was also performed in mice (Plan IV, Figure 4). In addition, a combined treatment with p.o. ascorbic acid (pre- and post-treatments) was performed for 10 days (250 mg/kg/day), beginning three days before radiation and lasting for seven days after radiation (Plan V, Figure 4). Furthermore, we added a group where the mice received all three treatments (p.o. administration of ascorbic acid for 10 days before/after radiation and the engulfment of ascorbic acid (250 mg/kg) eight hours before radiation) (Plan VI, Figure 4). Control mice received p.o. distilled water only.

### 3.4. Measurement of the Ascorbic Acid Levels in the Intestinal Tissue and Plasma

A sample of small intestine (approximately 0.7 g) was obtained from each mouse immediately after sacrifice (just before irradiation). After being washed with saline, each sample was homogenized in 5.4% metaphosphoric acid (9.8 g) and centrifuged at 10,000 × *g* for 15 min at 4 °C. Blood samples were obtained from the retro-orbital plexus in mice, and then plasma samples were collected by centrifugation. The ascorbic acid levels in the homogenate supernatant and plasma were measured by the SRL laboratory (Tokyo, Japan) using high performance liquid chromatography (HPLC, Shimadzu Co., Kyoto, Japan).

### 3.5. Pathological Examination of the Gastrointestinal Tract and Bone Marrow

Mice were euthanized to remove the gastrointestinal tract and the bone marrow, including that from the sternum, the lumbar vertebra and the femur. The gastrointestinal tract was immersed in 20% formalin for two days. The bone marrow was also immersed in 20% formalin for two days after three days of decalcification. Slides were prepared from the processed specimens and stained with hemotoxylin and eosin (H. E.).

### 3.6. Measurement of the Villus Height in the Intestine

The villus height was determined by measuring the distance from the base of the crypt to the villus tip in a longitudinal section of the intestine. For each mouse, 10–20 measurements of the villus height were obtained from three different tissue sections to calculate a mean height value. These mean values were then used for the statistical analysis.

### 3.7. TUNEL Staining of the Intestine

Mice were euthanized and sacrificed 12 h after radiation to obtain the small intestines for examination of the apoptotic cells, because radiation-induced apoptosis becomes evident at that time [33]. TUNEL (terminal deoxynucleotidyltransferase-mediated dUTP nick end labeling) staining was performed on tissue sections of formalin-fixed, paraffin-embedded mouse small intestine using an *in situ* apoptosis detection kit (MK500, Takara, Tokyo, Japan) [34,35]. Briefly, after deparaffinization, the slides were incubated with 10 mg/mL proteinase K at 37 °C for 15 min for antigen retrieval. Subsequently, specimen slides were placed in 0.3% H_2_O_2_ in methanol for five minutes. After they were washed with PBS, the slides were incubated with TdTenzyme at a 1:10 dilution in Labeling Safe Buffer in a moist chamber at 37 °C for 90 min. Then, slides were washed and incubated with anti-FITC HRP conjugate at 37 °C for 30 min. Reactions were visualized with 3,3′-diaminobenzidine (DAB). As a negative control, the slides were incubated with Labeling Safe Buffer alone.

### 3.8. Measurements of the Hematological Parameters, Plasma Citrulline Levels, Plasma and Intestinal Tissue Levels of Free Radical Metabolites and Proinflammatory Cytokines

Blood samples were obtained from the retro-orbital plexus of mice to measure the hematological parameters, such as the white blood cell (WBC) count, red blood cell (RBC) count, hemoglobin (Hb) levels and platelets using a hematology analyzer (PEC 170; Erma, Tokyo, Japan). The plasma citrulline levels were also measured using a fully automated amino acid analyzer (JLC-500/V2, Nihon Denshi, Tokyo, Japan). The tumor necrosis factor (TNF), interleukin (IL)-1β, and IL-6 levels in the plasma and intestinal tissue were measured using respective enzyme-linked immunosorbent assay (ELISA) kits (R & D Systems, Boston, MA, USA). The intestinal tissue protein concentrations were also assayed using a commercially available kit (Bio-Rad Laboratories, Richmond, CA, USA). Free radical metabolites in the plasma and intestinal tissue were measured using the d-ROMs test (Diacron, Grosseto, Italy) [4,36,37]. The d-ROMs test is a spectrophotometric method that assesses the overall oxidative stress by measuring the total hydroperoxide levels, because hydroperoxides are intermediate oxidative products of lipids, peptides and aminoacids. Briefly, 0.02 mL plasma was diluted in 1 mL acetate-buffered solution. Hydroperoxide groups react with the transition metal ions liberated from the proteins in the acidic medium, and are converted to alkoxyl and peroxyl radicals according to the Fenton reaction. These newly formed radicals, the quantities of which are directly proportional to those of the peroxides, are trapped chemically with 0.02 mL of a chromogen (*N*,*N*-diethyl-para-phenylendiamine), leading to the formation of the radical cation of this chromogen. The purple color resulting from this reaction over time can be monitored in a spectrophotometer (Wismarll FRAS4, Tokyo, Japan) at 505 nm. The results of this method are expressed in conventional units (Carratelli units [UCarr]). A sample of small intestine (50 mg) was obtained from the mice treated with or without ascorbic acid seven days after abdominal irradiation at 13 Gy. After being washed with saline, each sample was homogenized in 1 mL of phosphate buffered saline (PBS) and centrifuged at 10,000 × *g* for 15 min at 4 °C. The free radical metabolite levels in the homogenate supernatant were similarly measured using the d-ROMs test.

### 3.9. Statistical Analysis

The statistical analyses were performed using the Stat View 4.02J software package (Abacus Concepts, Berkeley, CA, USA). Survival rates were compared by the Wilcoxon signed rank test. Statistical evaluations were performed using one-way ANOVA, followed by the Bonferroni *post-hoc* test. The data are presented as the means ± SE. Values of *p* < 0.05 were considered to be statistically significant.

## 4. Conclusions

Combination therapy with ascorbic acid drastically improved mouse survivals after abdominal radiation, because it prevented lethal gastrointestinal damage. This combination therapy with ascorbic acid may be a potent therapeutic tool for radiation-induced gastrointestinal damage.

## Supplementary Information



## Figures and Tables

**Table 1:** Ascorbic acid levels in the small intestine and plasma after the treatments with ascorbic acid.

Group	Ascorbic acid levels	

	Tissue level of the small intestine (mg/L)	Plasma (mg/L)
No treatment with ascorbic acid	22.8 ± 2.0	5.2 ± 0.5
Engulfment (8 h before) with ascorbic acid	27.1 ± 1.4	5.2 ± 0.9
Pretreatment (for 3 days) with ascorbic acid	30.5 ± 1.6 †	6.2 ± 0.7
Pretreatment (for 3 days) + engulfment (8 h before) with ascorbic acid	36.1 ± 1.7 *	6.3 ± 0.6

Mice received the indicated treatments with ascorbic acid. The small intestine and plasma were obtained from the mice just before radiation. Data are shown as means ± SE from *n* = 5 in each group.

* *p* < 0.01 *vs.* no treatment and engulfment (8 h) and *p* < 0.05 *vs.* pretreatment (3 days), and

† *p* < 0.01 *vs.* no treatment.

**Table 2:** The levels of free radical metabolites and cytokines in the intestine and plasma in mice seven days after radiation.

Free radical metabolites, cytokines	Group	Tissue levels of the small intestine	Plasma levels
Free radical metabolites (UCarr)	No treatment	30 ± 1	234 ± 39
	Treatment with ascorbic acid	24 ± 2 *	235 ± 45

TNF (tissue levels: pg/mg protein)	No treatment	57 ± 7	40 ± 18
(Plasma levels :pg/mL)	Treatment with ascorbic acid	41 ± 1 †	17 ± 10

IL-1β (tissue levels: pg/mg protein)	No treatment	258 ± 155	Not detected
(Plasma levels :pg/mL)	Treatment with ascorbic acid	274 ± 103	Not detected

IL-6 (tissue levels: pg/mg protein)	No treatment	179 ± 79	185 ± 113
(Plasma levels :pg/mL)	Treatment with ascorbic acid	122 ± 106	111 ± 90

Mice were treated with or without ascorbic acid. Seven days after abdominal irradiation at 13 Gy, the small intestine (50 mg) and plasma were obtained from the mice to examine the levels of free radical metabolites, TNF, IL-1β and LI-6. Data are shown as means ± SE from *n* = 5 in each group.

* *p* < 0.01,

† *p* < 0.05 *vs.* no treatment.
