# Supplementary material for: A Combination of Pre- and Post-Exposure Ascorbic Acid Rescues Mice from Radiation-Induced Lethal Gastrointestinal Damage
Source: Int J Mol Sci. 2013 Sep 27;14(10):19618–35. doi: 10.3390/ijms141019618 (PMC3821576; doi:10.3390/ijms141019618)
Supplement: Supplementary file 1 [file ijms-14-19618-s001.pdf]

Supplementary Information

Figure S1. The pathological findings in mice that received abdominal irradiation at 13 Gy. The stomach, duodenum, jejunum, cecum and colon were removed from the mice seven days after abdominal radiation at 13 Gy, or from the mice without radiation. The images shown are representative of each group ($n = 5$), ×40 H. E.

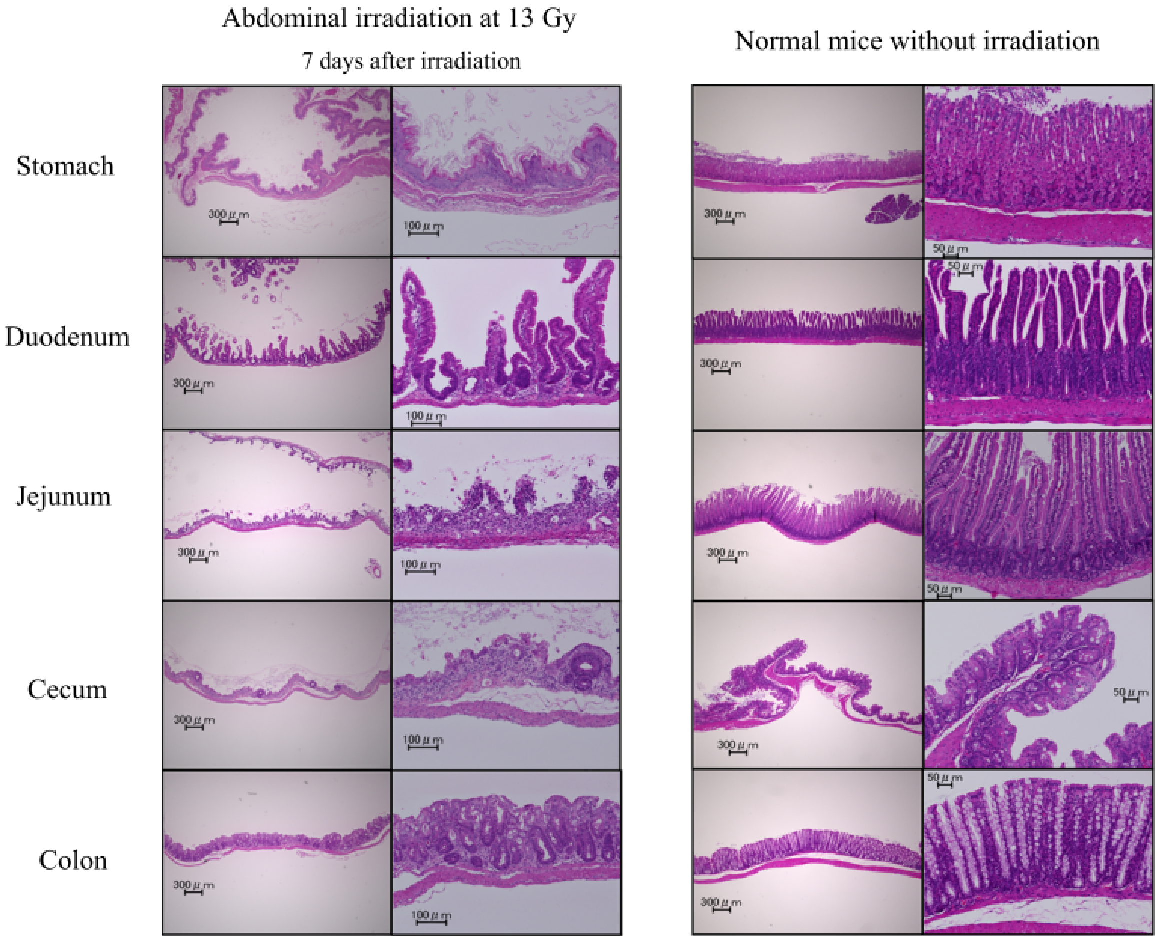

© 2013 by the authors; licensee MDPI, Basel, Switzerland. This article is an open access article distributed under the terms and conditions of the Creative Commons Attribution license (http://creativecommons.org/licenses/by/3.0/).
